# Induction of Fetal Hemoglobin *In Vivo* Mediated by a Synthetic **γ**-Globin Zinc Finger Activator

**DOI:** 10.1155/2012/507894

**Published:** 2012-06-15

**Authors:** Flávia C. Costa, Halyna Fedosyuk, Renee Neades, Johana Bravo de Los Rios, Carlos F. Barbas, Kenneth R. Peterson

**Affiliations:** ^1^Department of Biochemistry and Molecular Biology, University of Kansas Medical Center, 3901 Rainbow Boulevard, Kansas City, KS 66160, USA; ^2^Department of Molecular Biology and Chemistry, The Scripps Research Institute, La Jolla, CA 92037, USA; ^3^Department of Anatomy and Cell Biology, University of Kansas Medical Center, 3901 Rainbow Boulevard, Kansas City, KS 66160, USA

## Abstract

Sickle cell disease (SCD) and **β**-thalassemia patients are phenotypically normal if they carry compensatory hereditary persistence of fetal hemoglobin (HPFH) mutations that result in increased levels of fetal hemoglobin (HbF, **γ**-globin chains) in adulthood. Thus, research has focused on manipulating the reactivation of **γ**-globin gene expression during adult definitive erythropoiesis as the most promising therapy to treat these hemoglobinopathies. Artificial transcription factors (ATFs) are synthetic proteins designed to bind at a specific DNA sequence and modulate gene expression. The artificial zinc finger gg1-VP64 was designed to target the −117 region of the ^A^ **γ**-globin gene proximal promoter and activate expression of this gene. Previous studies demonstrated that HbF levels were increased in murine chemical inducer of dimerization (CID)-dependent bone marrow cells carrying a human **β**-globin locus yeast artificial chromosome (**β**-YAC) transgene and in CD34^+^ erythroid progenitor cells from normal donors and **β**-thalassemia patients. Herein, we report that gg1-VP64 increased **γ**-globin gene expression *in vivo*, in peripheral blood samples from gg1-VP64 **β**-YAC double-transgenic (bigenic) mice. Our results demonstrate that ATFs function in an animal model to increase gene expression. Thus, this class of reagent may be an effective gene therapy for treatment of some inherited diseases.

## 1. Introduction

Human hemoglobin is a tetrameric molecule composed of two *α*-like and two *β*-like chains, located on chromosomes 16 and 11, respectively. The *β*-like chain is comprised of the product of one of five functional genes (embryonic *ε*-, fetal ^A^ *γ*- and ^G^ *γ*-, and adult *δ*- and *β*-globin) which are developmentally expressed in the order that they are arrayed in the locus [1, 2]. As human erythroid development proceeds, the proper *β*-like globin genes are activated or repressed, giving rise to the different hemoglobin chains expressed throughout development [2]. Hemoglobin switching from fetal *γ*-globin to adult *β*-globin gene expression begins shortly before birth and is usually completed within the first 6 months after birth. In some individuals, hemoglobin switching is not completed, resulting in a condition called hereditary persistence of fetal hemoglobin (HPFH), which is characterized by high expression of fetal hemoglobin (HbF, *γ*-globin) during adult definitive erythropoiesis [1, 2]. Sickle cell disease (SCD) and *β*-thalassemia patients are phenotypically normal if they carry compensatory mutations that result in HPFH as well [1, 2]. These genetic studies have indicated that increased HbF will help alleviate pathophysiology associated with these hemoglobinopathies, and thus, research has focused on elucidating the pathways involved in the maintenance or activation of *γ*-globin expression by drug or gene therapy.

Pharmacological agents such as butyrate, decitabine, and hydroxyurea are effective in inducing HbF *in vitro* and *in vivo* [3]. To date, hydroxyurea, a ribonucleotide reductase inhibitor, is the only drug approved for clinical use in sickle cell patients [3]. Although it is effective in pediatric patients, the drug also has demonstrated effect on the induction of *γ*-globin in adult patients, but the long-term effect on organ damage, stroke, and carcinogenesis remains uncertain [3–5]. Thus, there is a need to develop new and more effective therapeutic drugs to treat SCD and *β*-thalassemia.

Many studies have demonstrated the role of stage-specific transcription factors in hemoglobin switching, indicating the potential therapeutic use of these transcription factors to treat hemoglobinopathies [6–9]. The zinc finger transcription factor *BCL11A* was recently shown to function as a repressor of HbF expression [6]. When erythroid Krüppel-like factor 1 (EKLF1, KLF1), an adult *β*-globin gene-specific zinc finger transcription factor, was knocked down in erythroid progenitor CD34^+^ cells, *γ*-globin expression was induced [9]. DRED (direct repeat erythroid definitive) is a repressor complex that binds to the direct repeat (DR) elements in the *ε*- and *γ*-globin gene promoters, and two of the components in this complex are the orphan nuclear receptors TR2 and TR4 [8]. Enforced expression of TR2/TR4 increased fetal *γ*-globin gene expression in adult erythroid cells from *β*-YAC transgenic mice [7] and also in adult erythroid cells from the humanized SCD mice [10]. These studies clearly demonstrate that manipulation of transcription factors efficiently reactivates *γ*-globin expression during adult definitive erythropoiesis.

The use of synthetic zinc finger transcriptional activators designed to interact with a specific DNA sequence and activate gene expression has been well documented [11–14]. In fact, data from studies in cell lines indicated that synthetic activators targeted to the proximal promoter of the ^A^ *γ*-globin gene have successfully induced *γ*-globin gene expression [11–15]. The artificial zinc finger gg1-VP64 was designed to interact with the −117 region of the ^A^ *γ*-globin gene proximal promoter [12]. A 7–16-fold increase in *γ*-globin expression was observed in K562 cells stably transfected with gg1-VP64 [12]. Increased *γ*-globin gene expression was also observed following transfection of the gg1-VP64 construct into immortalized bone marrow cells isolated from human *β*-globin locus yeast artificial chromosome (*β*-YAC) transgenic mice [11]. More recently, the gg1-VP64 activator was reported to significantly increase HbF levels in CD34^+^ erythroid progenitor cells from normal human donors and *β*-thalassemia patients [14, 15]. In this study we demonstrate that gg1-VP64 increased *γ*-globin gene expression during adult definitive erythropoiesis in *β*-YAC transgenic mice.

## 2. Materials and Methods

### 2.1. gg1-VP64 Construct 

Enforced erythroid-specific expression of the gg1-VP64-HA fusion, consisting of the gg1 zinc finger moiety, the VP64 activator, and an HA tag for detection of the protein fusion was obtained by cloning it into the unique *Bgl*II restriction enzyme site of p*μ*′LCR-*β* pr-*Bgl*II-*β* int2-enh, a vector previously shown to confer erythroid/megakaryocytic-restricted expression upon a linked gene [11, 12]. A 0.8 Kb *Apa*I-*Hind*III gg1-VP64 fragment was made blunt-ended and ligated into *Bg*lII-cut, blunt-ended, and phosphatased p*μ*′LCR-*β* pr-*Bgl*II-*β* int2-enh. Transgenic mice were generated as previously described [16, 17]. These mice were crossed to *β*-YAC transgenic mice [16] to produce four bigenic lines bearing the gg1-VP64 construct and a *β*-YAC reporter (2, 7, 10, and 18). PCR was employed to genotype the transgenic lines using the following primer sequences: *β*-YAC: Hu *ε*-globin forward, 5′-TTCTTGGAAAAGGAGAATGGGAGAGAT-3′; Hu *ε*-globin reverse, 5′-GCAGTAAAATGCACCATGATGCCAGGC-3′ and gg1-VP64: TF-3, 5′-TTCTCCCGCAGCGATCAC-3′ and TF-4, 5′-CCAAAGCACCTGGGTCTGA-3′ [12].

### 2.2. Phenylhydrazine Treatment of Mice 

Adult bigenic gg1-VP64 *β*-YAC and single transgenic *β*-YAC mouse lines at least 6 weeks old were given 60 mg phenylhydrazine (10 mg/mL in phosphate-buffered saline; P-6926; Sigma-Aldrich, St. Louis, MO, USA) per kg body weight via intraperitoneal injection for three consecutive days [18]. Mice were sacrificed 4 days posttreatment, and spleen, liver, and blood were harvested and processed for total RNA extraction and cellular lysate preparation.

### 2.3. Reverse-Transcriptase PCR (RT-PCR) and Real-Time Quantitative PCR (qPCR) 

Total RNA was prepared from adult blood and tissue lysates using the GenElute Mammalian Total RNA Purification Kit (Sigma-Aldrich, St. Louis, MO, USA).  cDNA was synthesized using the iScript cDNA Synthesis Kit (Bio-Rad, Hercules, CA, USA). RT-PCR was performed using gg1-VP64 specific primers TF-3, 5′-TTCTCCCGCAGCGATCAC-3′ and TF-4, 5′-CCAAAGCACCTGGGTCTGA-3′ [12].

qPCR analysis was performed with SYBR Green dye using MiniOpticon or CFX96 instruments (Bio-Rad, Hercules, CA, USA). Expression of *γ*- and *β*-globin was calculated using the relative quantification method, as previously described [19, 20], using samples from *β*-YAC transgenics as a control. PCR primer sequences utilized for expression studies were: Hu-*γ*1, 5′-GACCGTTTTGGCAATCCATTTC-3′; Hu-*γ*2, 5′-GTATTGCTTGCAGAATAAAGCC-3′; *β*-globin FWD, 5′-GAGAAGTCTGCCGTTACTGCC-3′; *β*-globin REV, 5′-CCGAGCACTTTCTTGCCATGA-3′; Mo-Gapdh FWD, 5′-AGGTTGTCTCCTGCGACTTCA-3′; Mo-Gapdh REV, 5′-CCAGGAAATGAGCTTGACAAAG-3′; Mo-*α*-globin FWD, 5′-GATTCTGACAGACTCAGGAAGAAAC-3′; Mo-*α*-globin REV, 5′-CCTTTCCAGGGCTTCAGCTCCATAT-3′. Triplicate data sets were generated, and qPCR results were normalized to murine Gapdh or *α*-globin genes.

### 2.4. Western Blot Analysis

Chemical inducer of dimerization (CID)-dependent *β*-YAC bone marrow cell [11] and CID-dependent gg1-VP64 *β*-YAC bone marrow cell lysates were prepared as described [21, 22]. Protein concentrations were measured spectrophotometrically using the Bradford assay. Fifteen *μ*g of cellular lysate was mixed with loading dye (50 mM Tris pH 6.8, 100 mM DTT, 2% SDS, 0.1% bromophenol blue, 10% glycerol) and heated at 95°C for 5 minutes, followed by separation in a 10% SDS-12% polyacrylamide gel using Tris-glycine buffer. Western blotting was performed as previously described [22], according to standard procedures [21].

### 2.5. Antibodies

Anti-*β*-actin (sc-21757 Santa Cruz Biotechnology, Santa Cruz, CA, USA) and anti-HA probe (Y-11, sc-805, Santa Cruz Biotechnology), goat anti-rabbit HRP (sc-2030, Santa Cruz Biotechnology), and goat anti-mouse HRP (sc-2031, Santa Cruz Biotechnology) antibodies were used for western blotting.

### 2.6. HbF Detection by Flow Cytometry

Detection of HbF (F cells) was performed by flow cytometric analysis. Briefly, mouse blood was collected from the tail vein in heparinized capillary tubes. Ten *μ*L of whole blood was washed in PBS and fixed in 1 mL 4% fresh paraformaldehyde (Sigma Aldrich, Saint Louis, MO, USA). The cells were centrifuged, the supernatant discarded, and the pellets were resuspended in 1 mL ice-cold acetone : methanol (4 : 1) for 1 minute. Cells were washed twice in ice-cold PBS/0.1% BSA and resuspended in 800 *μ*L of PBS/0.1% BSA/0.1% Triton X-100 (PBT). One *μ*g sheep anti-human hemoglobin F-FITC-conjugated antibody (A80-136F, Bethyl Laboratories, Montgomery, TX, USA) was added to 100 *μ*L of the cell suspension and incubated for 40 minutes at room temperature. Cells were washed twice with 1 mL ice-cold PBS/0.1% BSA, and the pellets were resuspended in 200 *μ*L of PBS.**  **Cells were analyzed using a BD LSRII (BD Biosciences, San Jose, CA, USA) with a 530/30 nm emission filter (FITC/GFP). Data from 30,000 events was acquired for analysis using BD FACSDiva software (BD Biosciences, San Jose, CA, USA).

## 3. Results

### 3.1. Establishment of gg1-VP64 *β*-YAC Transgenic Lines

**Figure 1:**
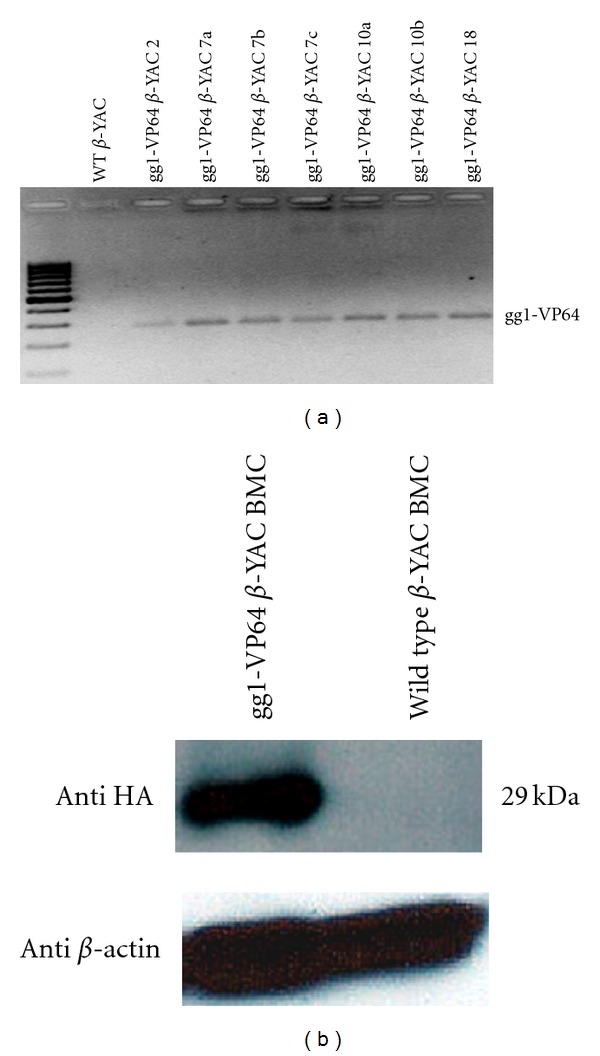
Expression of gg1-VP64. (a) Total RNA isolated from gg1-VP64 *β*-YAC bigenic line adult peripheral blood was analyzed by RT-PCR using gg1-VP64-specific primers. Each lane shows an individual from the established lines; numbers are indicated at the top of the panel; the gg1-VP64 product is indicated to the right side of the panel. (b) Cellular lysates from CID-dependent gg1-VP64 *β*-YAC BMCs were assayed by western blotting using an anti-HA tag antibody to detect the gg1-VP64-HA fusion (29 kDa, indicated to the right of the panel). CID-dependent *β*-YAC BMCs were used as the negative control. Anti-*β*-actin was employed as loading control. M, marker lane.

To evaluate the effect of the synthetic zinc finger gg1-VP64 on *γ*-globin gene expression during adult definitive erythropoiesis, gg1-VP64 transgenic lines were produced and bred to *β*-YAC transgenic mice [16, 17, 23]. Four gg1-VP64 *β*-YAC bigenic lines were obtained (lines 2, 7, 10, and 18), and samples from these lines were utilized in this study. The presence of the gg1-VP64 construct was confirmed by the presence of a PCR product amplified from a specific region of the gg1-VP64 construct. In addition, the presence of the human *β*-globin locus was confirmed by PCR amplification of the human *ε*-globin gene, to confirm the presence of the *β*-YAC transgene (see the Materials and Methods section). Expression of gg1-VP64 in adult blood samples of the gg1-VP64 *β*-YAC bigenic lines at the mRNA level was confirmed by RT-PCR (Figure 1(a)). Amplification of the gg1-VP64 fragment was observed exclusively in samples containing the gg1-VP64 construct.

To further demonstrate expression of the gg1-VP64 fusion at the protein level, CID-dependent BMCs were derived from gg1-VP64 *β*-YAC bigenic mice as previously described [11]. These BMCs maintained the same globin gene expression pattern observed in the adult transgenic mice. Western blotting was performed using an anti-HA tag antibody, which specifically recognizes the HA tag in the gg1-VP64 construct utilized to generate the transgenic lines [12]. A 29 KDa fragment corresponding to the HA-tagged gg1-VP64 fragment was detected in the gg1-VP64 *β*-YAC CID BMCs, but not in *β*-YAC CID BMCs lacking gg1-VP64 used as the control (Figure 1(b)). Together, these data confirm the expression at the protein level of the gg1-VP64 zinc finger construct in the gg1-VP64 *β*-YAC bigenic lines.

### 3.2. Expression of Fetal Hemoglobin in gg1-VP64 *β*-YAC Mice during Adult Definitive Erythropoiesis

**Figure 2:**
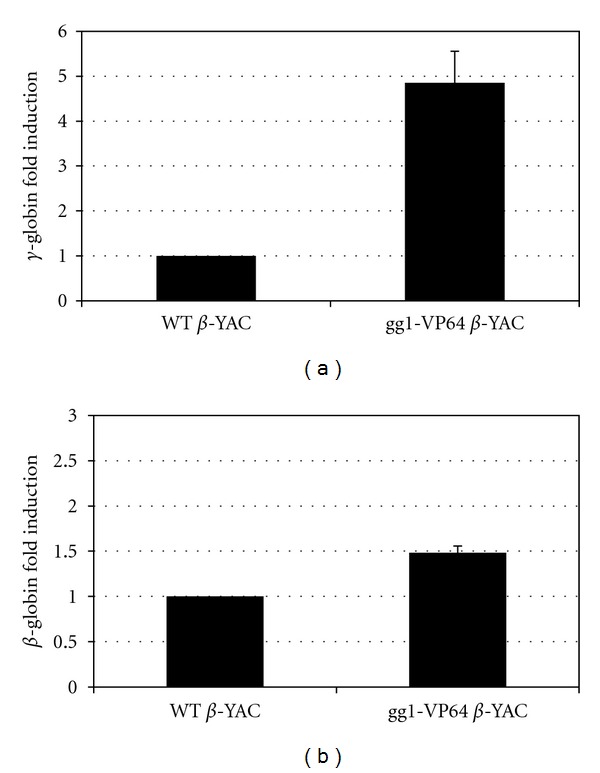
Human *β*-like globin gene expression in adult blood samples from gg1-VP64 *β*-YAC transgenic mice. Total RNA isolated from adult blood was subjected to qPCR analysis using SYBR Green. Primers for human *γ*- and *β*-globin were utilized, and the data was normalized to mouse *α*-globin or Gapdh gene expression. (a) *γ*-globin gene expression. (b) *β*-globin gene expression. Results are the average of 7 different gg1-VP64 *β*-YAC bigenic mice ± the standard error of the mean (SEM). Student's *t*-test values were *P* < 0.01 for *γ*-globin and *P* > 0.1 for *β*-globin.

To test whether the presence of gg1-VP64 induced *γ*-globin expression during adult erythropoiesis in *β*-YAC transgenic mice, human *β*-like globin gene expression was measured by qPCR in adult blood from F_2_ or F_3_ generation adult mice. Mouse *α*-globin and Gapdh served as internal controls to quantitate human *β*-like globin transgene expression levels. All values were normalized to these internal controls and corrected for transgene and endogenous gene copy number. A 5-fold increase in *γ*-globin gene expression was observed in the peripheral blood samples from the gg1-VP64 *β*-YAC bigenic line compared to the wild-type *β*-YAC mice (Figure 2(a)). The expression of the adult *β*-globin gene was demonstrated to be slightly increased in the adult blood samples from the gg1-VP64 *β*-YAC bigenic lines, but this increase was not significant (Figure 2(b)).

**Figure 3:**
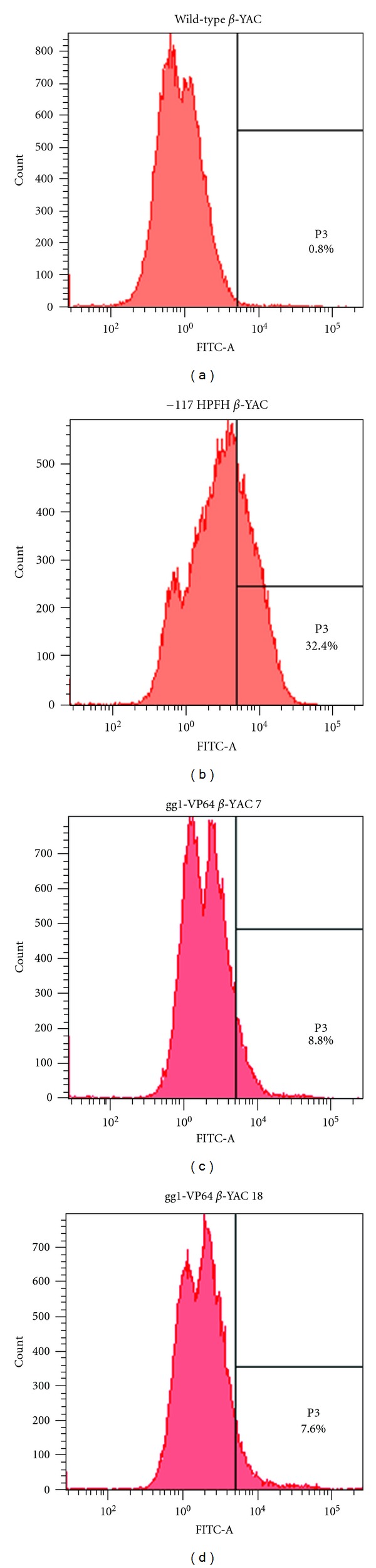
Flow cytometry analysis of F cells in blood from two adult gg1-VP64 *β*-YAC bigenic mice. A sheep anti-human hemoglobin F-FITC-conjugated antibody was used to determine the percentage of HbF-expressing cells. (a) wild-type *β*-YAC; (b) −117 Greek HPFH *β*-YAC; (c) gg1-VP64 *β*-YAC 7; (d) gg1-VP64 *β*-YAC 18.

**Figure 4:**
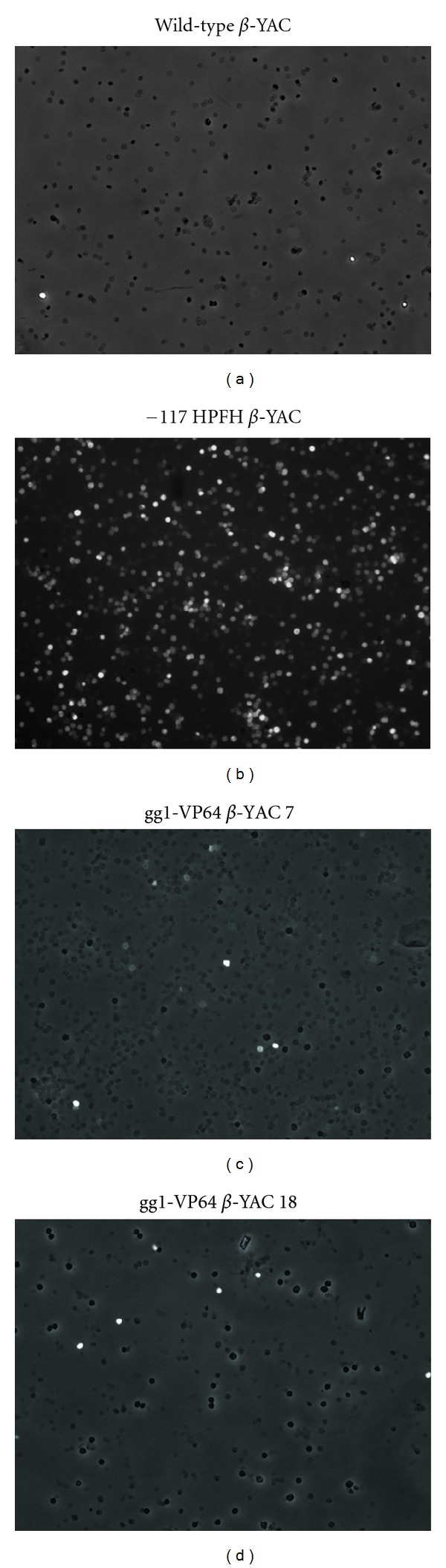
Staining of gg1-VP64 *β*-YAC bigenic mouse adult blood with anti-human hemoglobin F-FITC-conjugated antibody. Processing of peripheral blood cytospins was performed as described in the Materials and Methods section. (a) Wild-type *β*-YAC; (b) −117 Greek HPFH *β*-YAC; (c) gg1-VP64 *β*-YAC 7; (d) gg1-VP64 *β*-YAC 18.

To further demonstrate that increased *γ*-globin mRNA expression in the gg1-VP64 *β*-YAC bigenic lines correlates with an increased percentage of HbF-containing cells, flow cytometry analysis was performed using an anti-human hemoglobin F-FITC-conjugated antibody. The gg1-VP64 *β*-YAC bigenic mice showed an 8.8% and 7.6% increase of F cells (Figures 3(c) and 3(d)) compared to a wild-type *β*-YAC transgenic control (0.8% F cells; Figure 3(a)). Positive controls included the previously characterized −117 Greek HPFH *β*-YAC mice (32.4% F cells; Figure 3(b)). We also performed staining of gg1-VP64 *β*-YAC bigenic mouse peripheral blood cytospins with the same antibody (Figure 4), which demonstrated a heterocellular distribution of F cells in the gg1-VP64 *β*-YAC animals (Figures 4(c) and 4(d)), compared to a pancellular distribution in −117 Greek HPFH *β*-YAC mice (Figure 4(b); [23]). Although only one representative microscope field is shown in each panel of Figure 4, the number of positively stained cells was approximately 10-fold higher compared to wild-type *β*-YAC transgenic mice (Figure 4(a); data not shown).

**Figure 5:**
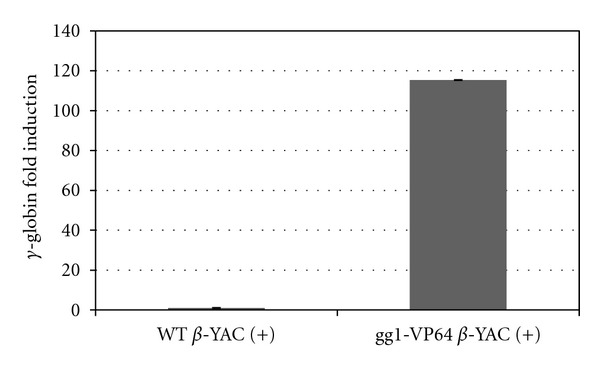
*γ*-globin gene expression in phenylhydrazine-treated samples from gg1-VP64 *β*-YAC bigenic mice. Total RNA isolated from adult spleen was subjected to qPCR analysis using SYBR Green. Primers for human *γ*-globin were utilized, and the data was normalized to mouse *α*-globin or Gapdh gene expression. Results are the average of 3 replicates of the gg1-VP64 *β*-YAC bigenic mouse ± the standard error of the mean (SEM).

 The effect of gg1-VP64 was also assessed in RNA samples extracted from spleens of phenylhydrazine-treated gg1-VP64 *β*-YAC bigenic mice. Phenylhydrazine treatment induces high levels of *γ*-globin gene expression due to the reticulocytosis resulting from hemolytic anemia [18]. qPCR was performed on RNA samples from gg1-VP64 *β*-YAC line 7, and a 100-fold increase in *γ*-globin expression was observed compared to the phenylhydrazine-treated *β*-YAC control mice (Figure 5). Together our data demonstrate that the zinc finger gg1-VP64 construct increased *γ*-globin gene expression *in vivo* during adult definitive erythropoiesis.

## 4. Discussion

The use of synthetic gene-targeted transcription factors that bind to specific DNA sequences to regulate the expression of endogenous genes is an emerging field. Engineered zinc finger transcription factors in which zinc finger motifs are coupled to an activation domain provide new therapeutic venues to enhance gene expression and treat diseases such as hemoglobinopathies [14, 15, 24–26].

The transcription factor gg1-VP64 is a hexameric zinc finger-based DNA binding domain, designed to interact specifically with an 18-base pair target DNA sequence at the −117 nucleotide in the proximal promoter of the ^A^ *γ*-globin gene [12]. Our study demonstrates increased *γ*-globin gene expression at both the mRNA and protein level *in vivo* during adult definitive erythropoiesis in gg1-VP64 *β*-YAC transgenic mice. Our data corroborate previously published data where *γ*-globin gene expression is increased in K562 cells, in CID-dependent *β*-YAC BMCs and human erythroid CD34^+^ progenitor cells following transfection of the gg1-VP64 construct [11–15]. A G-to-A mutation at position −117 of the ^A^ *γ*-globin gene is associated with high levels of fetal hemoglobin in the Greek population (Greek hereditary persistence of fetal hemoglobin or HPFH) [27]. This mutation alters a direct repeat element (DR1) in the ^A^ *γ*-globin gene promoter [7, 8, 28]. Interestingly, a complex called DRED (direct repeat erythroid-definitive) binds this same region, silencing the fetal *γ*-globin gene [7].

Many studies have been performed in transgenic mouse models bearing human *β*-globin locus constructs [16, 29–31]. Unlike humans, mice do not have a fetal-stage-specific hemoglobin. However, the human ^A^ *γ*-globin gene functions as a fetal gene in mice, and the HPFH phenotype is recapitulated in transgenic mice containing −117, −175, −195, or −566 ^A^ *γ*-globin HPFH point-mutant globin constructs or *β*-YACs ([23, 27, 32–34], unpublished data). These models have been utilized extensively to understand the function of *cis*-acting elements and *trans*-acting factors within the *γ*-globin locus, including their potential effects in restoring *γ*-globin expression in adult erythropoiesis [23, 27, 32–34]. Recently, enforced expression of the *trans*-acting factor TR2/TR4 orphan nuclear receptor was shown to increase *γ*-globin gene expression in adult erythroid cells of the humanized SCD mouse model [10]. In another study, knockout of *BCL11A* in SCD mice was shown to increase *γ*-globin expression and red cell survival, thus correcting the SCD phenotype [35]. Taken together, these studies demonstrate the utility of mouse models for screening transcription factors that can reactivate HbF *in vivo*. Finally, the data presented in this study indicates that a synthetic transcription factor can induce the expression of *γ*-globin gene expression and HbF *in vivo* during adult definitive erythropoiesis in transgenic mice and supports the use of these constructs as a potential new therapy to treat sickle cell disease and other hemoglobinopathies.
